# Photoisomerization of phytochrome's chromophore: a vibrational spectroscopic view on the primary ground state processes

**DOI:** 10.1039/d5ra08731g

**Published:** 2025-12-15

**Authors:** Galaan Merga, Maximilian Große, Patrick Piwowarski, Anastasia Kraskov, Francisco Velazquez Escobar, Norbert Michael, Manal Ebrahim, Luisa Sauthof, Patrick Scheerer, Franz Bartl, Peter Hildebrandt

**Affiliations:** a Humboldt-Universität zu Berlin, Institut für Biologie, Biophysikalische Chemie Invalidenstr 42 D-10115 Berlin Germany Franz.Bartl@HU-Berlin.de; b Technische Universität Berlin, Institut für Chemie Sekr. PC14, Straße des 17. Juni 135 D-10623 Berlin Germany Peter.Hildebrandt@TU-Berlin.de; c Institute of Medical Physics and Biophysics, Group Structural Biology of Cellular Signaling Charité – Universitätsmedizin Berlin, Corporate Member of Freie Universität Berlin and Humboldt-Universität zu Berlin Charitéplatz 1 D-10117 Berlin Germany

## Abstract

The function of the biological photoswitch phytochrome is initiated by photoisomerization of the methine-bridged tetrapyrrole chromophore, followed by thermal relaxation steps. As a result of this reaction cascade, the protein interconverts between two parental state. These states, denoted as Pr (red absorbing) and Pfr (far-red absorbing), represent the physiologically inactive and active form of the protein, respectively. In this work we studied the primary photoprocesses of two bacterial phytochromes Agp1 and Agp2, in which either Pr or Pfr is the stable dark state, respectively. We employed cryogenic IR difference and resonance Raman spectroscopy between 4 K and 130 K to trap and characterize the species formed on the reaction pathways from Pfr to Lumi-F in Agp2 and Pr to Lumi-R in Agp1. The spectra analysis primarily focuses on the C=O stretching modes, which are assigned based on isotopic labelling experiments. In both proteins, three sub-states were identified, which reveal similar patterns of sequential structural changes. In the first sub-state L1 of both photoreceptors, generated at 4 K, structural changes are restricted to the isomerization site including rings D and C. In L2, formed at 30 K in Agp2 but at the same temperature range with L1 in Agp1, the structural changes propagate to ring B, and in L3 also include ring A. Comparison with previously published studies demonstrates that the present approach of cryogenic vibrational spectroscopy provides important structural insights that complement results from crystallography and ultrafast time-resolved spectroscopy.

## Abstract 2

Photoisomerization of the parent states of phytochromes were investigated by cryogenic vibrational spectroscopy revealing the stepwise evolution of structural changes from the isomerization site through the entire tetrapyrrole chromophore.

## Graphical abstract

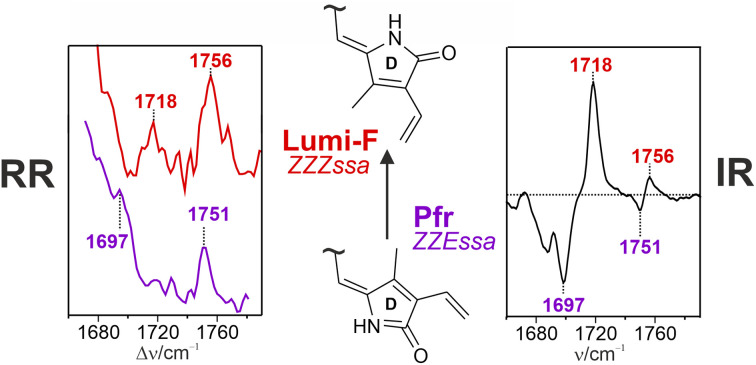

## 1. Introduction

**Fig. 1:**
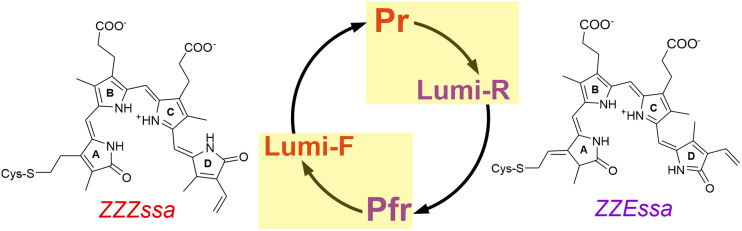
Structural formulas of the biliverdin (BV) chromophore in the *ZZZ*ssa (left) and *ZZE*ssa (right) configuration. The states with a *ZZZ*ssa and *ZZE*ssa BV are indicated in red and violet, respectively. A simplified view of the photocycle is shown in the middle (a more detailed presentation is given in the SI, Fig. S1). The photochemical reactions are highlighted by yellow boxes. The other reaction arrows represent series of thermal reactions including the *meta*-R and *meta*-F intermediates.

Phytochromes are biological photoswitches found in plants, fungi, and bacteria that interconvert between the parent states Pr and Pfr (Fig. 1).^1–4^ These states are associated with an active and inactive output module, which in many cases is a histidine kinase. (De)activation of the output module that initiates physiological processes is based on a series of hierarchical structural changes, starting with the *Z*/*E* photoisomerization of the bilin chromophore either in the *ZZZ*ssa (Pr) or *ZZE*ssa (Pfr) configuration.^5^ The photoreaction is followed by thermal relaxation steps, initially restricted to the chromophore and its immediate environment. The structural changes then stepwise extend to protein regions remote from the chromophore binding pocket and comprise the secondary and tertiary protein structure. Eventually they are transmitted to the output module.^2^

Many studies have focused on the primary photoprocesses of phytochromes of both Pr and Pfr.^6–33^ However, despite the immense effort, details of the mechanism are still elusive. Moreover, molecular pathways may be different for the Pr → Lumi-R and Pfr → Lumi-F reactions. For the *Z* → *E* isomerization in Pr, an intermediate in the excited state was detected by femto-second (fs) stimulated resonance Raman (FSRS).^17^ In addition, more than one excited state intermediate were postulated in bacterial phytochromes to account for the distribution of reaction rates,^17,18^ which, however, was meanwhile shown not to be related to structural heterogeneity.^20^ In contrast, for the cyanobacterial phytochrome Cph1, two distinct Pr ground state conformations were identified and only one of them was photochemically competent.^34^ The photodynamics of the Pfr-to-Lumi-F reaction, *i.e.* the *E* → *Z* isomerization, was found to be faster than the reverse *Z* → *E* process of Pr, presumably due to a lower energy barrier between the Franck–Condon (FC) and conical intersection (CI) region.^35^

Investigation of the initial events of these photoconversions, *i.e.* the processes in the excited state and the decay to the Lumi intermediates in ground state, requires ultrafast spectroscopic techniques.^6,17,23,35^ Conversely, the subsequent relaxations in the ground state of the Lumi intermediates, which are in the focus of the present study, can also be monitored by cryogenic vibrational spectroscopy. This method has been widely applied down to temperatures of *ca.* 90 K using resonance Raman (RR) and infrared (IR) difference spectroscopy.^16,19,36,37^

In this work, we have extended the investigations to temperatures below 90 K to trap potential ground state precursors of the Lumi-R and Lumi-F states, which in turn are typically stable up to 150 K or even higher.^19^ We have studied the photoconversions in two phytochromes from *Agrobacterium fabrum*,^38–40^*i.e.*, Pfr to Lumi-F (*E* → *Z*) of the bathy phytochrome Agp2, and Pr to Lumi-R (*Z* → *E*) of the prototypical phytochrome Agp1. In Agp2 and Agp1, Pfr and Pr are the stable dark states, respectively. We analyzed the vibrational spectra with particular emphasis on the C=O stretching modes of the biliverdin (BV) chromophore (Fig. 1), that give rise to strong signals in IR spectroscopy which are, albeit significantly weaker, also detectable in RR.^37^ The present study identifies three sub-states of the ground state photoproducts, trapped between 4 and 130 K. The results are discussed in the context of previously published transient IR spectroscopic data.

## 2. Experimental section

### 2.1. Protein expression and purification

The full-length Agp1 and photosensory core module Agp2 were expressed and purified as described previously.^41–44^ For spectroscopic experiments, dark-adapted protein was concentrated to *ca.* 1 mM for RR and IR spectroscopy. The solution was buffered with Tris (pH 7.8). H/D exchange was achieved *via* an Amicon® ultracentrifugal filter by washing the sample five times with the D_2_O buffer (pD 7.8). For Agp2, this procedure was carried out in the dark to keep the protein in the Pfr state (H/D exchange only at rings A, B, and C). For a complete H/D exchange, the partially deuterated Pfr was converted to Pr and then back to Pfr.

### 2.2. IR difference spectroscopy

For IR measurements, the protein samples were placed in sample holders with a 6 mm PTFE-spacer. In cryogenic IR experiments, the sample was cooled to the desired temperature with an OptistatTN cryostat (Oxford Instruments). The spectrum was recorded using a Bruker IFS66v/s spectrometer (2 cm^−1^ spectral resolution) equipped with a mercury cadmium telluride (MCT) detector (J15D series, EG&G Judson). The light-induced spectrum was recorded during irradiation with 685 nm for *ca.* 60 s. The difference spectra calculated from the single-channel spectra before and after irradiation were pre-processed with the OPUS 7.5 software package (Bruker Optics, Karlsruhe, Germany). Whereas for Agp2 high-quality spectra could be obtained in the entire temperature range under consideration, all attempts failed to generate evaluable difference spectra for Agp1 between 40 and 120 K. In this temperature range, only in the C=O stretching region and only for H_2_O, weak signals could be identified.

### 2.3. Resonance Raman spectroscopy

RR measurements were performed based on a Bruker Fourier-transform MultiRAM spectrometer with a Ramanscope III. A Nd-YAG continuous-wave laser with a line width of 1 cm^−1^ was used for excitation at 1064 nm. The experiments were carried out with a nitrogen-cooled cryostat from Resultec (Linkam). Spectra of the samples in frozen solution (*ca.* 1 mM, pH 7.8 or pD 7.8) were recorded at *ca.* 120 K (Agp1) or 80 K (Agp2) with a laser power of 680 mW at the sample and an accumulation time of typically one hour. Potential laser-induced damage of the phytochrome samples could be ruled out, since comparison of RR spectra before and after a series of measurements did not reveal any differences. For the photoconversion, the protein sample was illuminated with a 780 nm laser diode for 3–5 minutes. Residual contributions from the parent state were removed by manually weighted spectra subtraction using the OPUS software (Bruker).

## 3. Results and discussion

The reactions Pfr → Lumi-F of Agp2 and Pr → Lumi-R of Agp1 were studied using cryogenic IR and RR spectroscopy, focusing on the C=O stretching modes. Thus, the analysis starts with an assignment of these modes for the parent states (Pfr, Pr) and the conformationally relaxed photoproducts (Lumi-F, Lumi-R), which are stable between 130 and 180 K. In the second step, cryogenic IR difference spectroscopy, supported by RR spectroscopy, was carried out over a wide temperature range from 4 K to 130 K to identify possible sub-states of the Lumi intermediates. The results are compared with previously reported data obtained by transient IR spectroscopy at ambient temperature in the picosecond (ps) time range.

### 3.1. Vibrational assignments

The vibrational assignments of the chromophore in the parent states Pr and Pfr were discussed in detail previously.^45–48^ Extending these assignments to Lumi-R and Lumi-F is difficult in view of the distorted chromophore structures.^19^ Here, we rely on previous analyses of the Lumi-R state^19^ and Lumi-F^49^ which employed isotopically labelled chromophores. In this work, special attention is laid on the C=O stretching modes of BV which serve as important spectral markers for changes of the structure of the terminal pyrrole rings of the BV cofactor and the immediate molecular environment. These modes include contributions from the N–H bending coordinates of the same ring which accounts for their H/D sensitivity. Due to non-covalent interactions of both the C=O and the N–H groups with adjacent water molecules and amino acid residues, these modes are sensitive indicators for conformational sub-states in the chromophore binding pocket, which may result in more than one C=O stretching mode of ring A and D, as shown previously.^50^

**Fig. 2:**
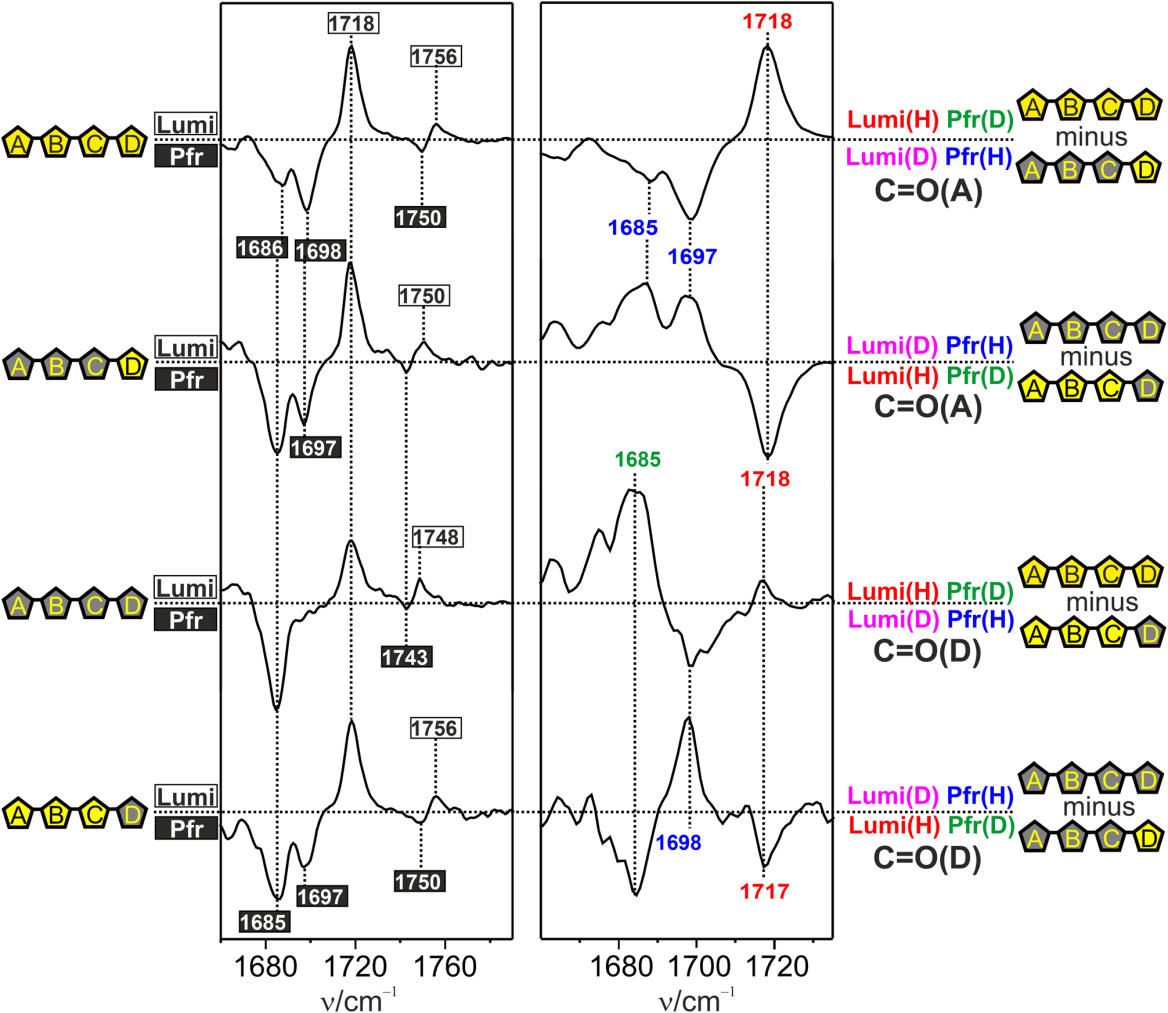
IR difference spectra of the Pfr → Lumi-F transition of Agp2 in the C=O stretching region, measured at 130 K. Left panel, “Lumi-F” minus “Pfr” difference spectra with the bands of Lumi-F and Pfr labeled by black numbers in white boxes and white numbers in black boxes, respectively. The spectra were measured from states of various H/D patterns of the BV N–H groups as indicated by the symbols of the left side. The letters denote the four pyrrole rings (A, B, C, D) with the nitrogens carrying a proton (yellow pentangle, black letter) or deuteron (gray pentangle, yellow letter). The different H/D patterns were generated by sequential H/D exchange as described in the text and in our previous work.^51^ Right panel, double difference spectra using the difference spectra from the left panel, and characterized by the pentangle symbol scheme (right side). The double difference spectra in this spectral region mainly reflect C=O(A) or C=O(D) signals as noted on the right side. The assignment of the respective positive and negative signals is illustrated by a color code with blue, green, red, and magenta referring to Pfr(H), Pfr(D), Lumi-F(H), and Lumi-F(D), respectively. The same color code also holds for labeling of the signals. The horizontal dotted lines mark the intensity difference of zero. The double difference spectra are scaled to optimize the visibility of the difference signals. Compared to the spectra on the left panel, this scaling resulted in an amplification of the signal intensities by a factor of *ca.* 1.33 and 2.25 for the top two and bottom two spectra, respectively.

In Agp2 the vibrational assignment of the C=O stretching modes is facilitated by the possibility of selective H/D exchange at the pyrrole N–H groups of BV.^51,52^ This allows for a discrimination of the C=O stretching modes of ring A and D. The selective H/D exchange is a characteristic property of bathy phytochromes that results from the tight salt bridge of the ring D N–H group with the side chain of the highly conserved Asp196.^52^ Thus, H → D exchange in the dark (Pfr) only leads to the replacement at the N–H groups of ring A, B, and C as well as the protonated propionic side chain of ring C (propC). After photoisomerization, the ring D N–H–Asp196 salt bridge is broken but only upon formation of Pr the exchange also occurs at ring D. The D → H back exchange follows the same scheme, which altogether results in four different H/D substitution patterns (Fig. 2). Due to the coupling of the adjacent N–H bending coordinate, the C=O stretching modes of ring A and D specifically respond to the selective H/D substitutions of ring A and D. Thus, the C=O stretching modes could be assigned for Pfr, *meta*-F, and Pr,^51^ although for Pfr some revisions will be made in the present study.

Fig. 2 (left panel) shows the “Lumi-F” minus “Pfr” IR difference spectra at different H/D patterns. Most remarkably, there is only a single positive Lumi-F peak that remains at the same frequency (1718 cm^−1^) regardless of the H/D pattern such that, solely based on these spectra, an assignment of the C=O stretching modes of ring A and D is not possible.

Conversely, the H/D sensitivity of the protonated propC C=O stretching shows the known behavior for Lumi-F and Pfr since its proton is exchanged already in the dark.^51^ However, as for Lumi-F, the assignment of the carbonyl stretching modes of Pfr is not straightforward. In the fully protonated difference spectrum, there are two negative Pfr signals at 1687 and 1698 cm^−1^, which in the fully deuterated state are replaced by a single negative band at 1685 cm^−1^. These findings imply that the C=O modes of both ring A and D appear at the same frequency in the fully deuterated state. To assign the bands in the protonated state, we now turn to spectra of states with selective exchange at ring A or ring D. In both cases, we note two negative Pfr signals at frequencies very similar to the fully protonated state. Thus, we conclude that the carbonyl function at ring A or D or both adopt two sub-states with frequencies at *ca.* 1698 and 1685 cm^−1^ in the fully protonated state.

**Fig. 3:**
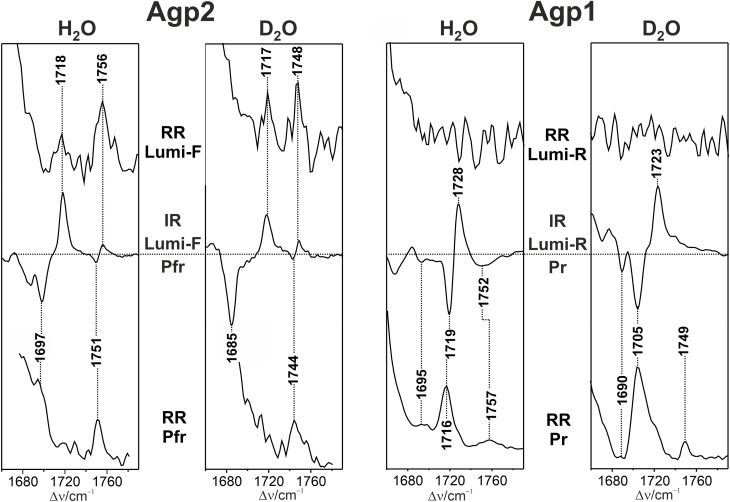
RR and IR spectra of Agp2 and Agp1 in H_2_O and D_2_O. The spectra were obtained at 130 K except for the IR spectrum of Agp1 in D_2_O which was measured at 150 K. The horizontal dotted line in the IR difference spectra mark the intensity difference of zero.

We then used these spectra to construct double difference spectra that separate signals from the two C=O stretching modes (Fig. 2, right panel). Thus, two of these spectra display the C=O stretching signals of ring A, with the conjugate pairs “protonated Lumi-F”/“deuterated Pfr” and “deuterated Lumi-F”/“protonated Pfr”, and the other two spectra show the same pairs for the C=O stretching of ring D. Hence, two bands at 1685 and 1697 cm^−1^ can readily be assigned to the C=O(A) modes of two sub-states of Pfr in the protonated form which upon deuteration coincide at 1685 cm^−1^. This signal overlaps with the low frequency component of C=O(A) of opposite sign. For Lumi-F, only one mode is detected at 1718 cm^−1^ that exhibits no H/D dependent shift. Similarly, the double difference spectra of the C=O(D) mode reveal one signal at 1698 cm^−1^ in the protonated state that shifts to 1685 cm^−1^ upon deuteration at ring D. However, neither the single (Fig. 2, left panel) nor the double difference spectra (Fig. 2, right panel) rule out that an additional C=O(D) conformer of Pfr with a frequency at 1685 cm^−1^ (as for C=O(A)) coexists with that at 1698 cm^−1^. In fact, temperature dependent studies show (*vide infra*) that the 1685 cm^−1^ signal prevails at very low temperatures but becomes very weak upon approaching 130 K. At this temperature, Lumi-F shows only the H/D insensitive band at 1717 cm^−1^. The assignments for the ring D C=O stretching are in line with the RR spectra (Fig. 3).

Due to the lower resonance enhancement the C=O mode of ring A cannot be identified in the RR spectra of Pfr and Lumi-F, whereas the RR activity of corresponding mode of ring D is sufficient for detection. Note that there are slight deviations between the frequencies of the RR and IR signals which mainly results from the difference formation in the latter case. In case of discrepancies, the assignments listed in Table 1 thus report the RR frequencies if the S/N ratio is sufficient for a reliable peak maximum determination. The RR spectra of Pfr and Lumi-F with different H/D patterns are shown in the SI (Fig. S2–S4).

**Table 1:** C=O stretching mode frequencies (in cm^−1^) of Agp2 (Pfr, Lumi-F) and Agp1 (Pr, Lumi-R) from cryogenic spectroscopy

	State	Sub-state^d^	C=O(A)		C=O(D)		propC	
			H_2_O	D_2_O	H_2_O	D_2_O	H_2_O	D_2_O
Agp2	Pfr	I, 130 K	1697^a^	1685^a^	1697^a^	1684^a^	1751^a^	1744^a^
		II, 130 K	1685^a^	1685^a^	1685^a^	1685^a^		
	Lumi-F	L1, 4–30 K			1694^b^			
		L2, 30–90 K			1707^b^		1745^b^	1737^b^
		L3, 130 K	1718^b^	1718^b^	1718^b^	1717^b^	1756^b^	1748^b^
Agp1	Pr^a^	I, 130 K	1732^c^	1721^c^	1716^c^	1705^c^		
		II, 130 K	1757^b^	1749^b^	1695^c^	1690^c^		
	Lumi-R	L1, 4–30 K			1697^b^			
		L2, 4–30 K			1706^b^			
		L3, 130 K			1728^b^	1723^b^		

a Assignments taken from previous work.^51^ Minor differences between the reported and the listed frequencies are due to the different temperatures.

b This work.

c Assignments taken from previous work.^50^ Minor differences between the reported and the listed frequencies are due to the different temperatures.

d The nomenclature L1, L2, and L3 is adopted from the study by Yang *et al.*^55^

Now we turn to the Pr and Lumi-R states of Agp1. Unlike Agp2, a selective H/D exchange is not possible for Agp1. For Pr, a plausible assignment of the C=O modes is based on previous studies on Agp1 and related prototypical phytochromes^48,50,53,54^ (Table 1).

For C=O(D), there are two bands at 1695 and 1716 cm^−1^, which in D_2_O shift down to 1690 and 1705 cm^−1^, respectively (Fig. 3). Note that the 1695 cm^−1^ band is largely obscured in the Lumi-R/Pr IR difference spectrum. Instead, it can readily be seen in the Pfr/Pr IR difference spectrum (SI, Fig. S5).^50^ Also the corresponding ring A C=O stretching at 1732 (1721) cm^−1^ in H_2_O (D_2_O) can only be identified in the Pfr/Pr IR difference spectrum.^50^ In Lumi-R, only one ring D C=O stretching was detected in the cryogenically trapped reference state (130–150 K) with a frequency of 1728 and 1723 cm^−1^ in H_2_O and D_2_O, respectively (Fig. 3). Unfortunately, the resonance enhancement of the C=O stretchings is too weak in Lumi-R to identify a band in this region at all. In contrast, the RR spectrum of Pr reveals distinct peaks in both H_2_O and D_2_O which confirms the assignments derived from IR difference spectra.^50^ Moreover, the RR spectrum shows an additional band at 1757 cm^−1^ in H_2_O, which seems to be related to the broad negative signal in the Lumi-R/Pr spectrum at 1752 cm^−1^. The Raman band displays a distinct downshift to 1749 cm^−1^ in D_2_O. These frequencies are very similar as those of the C=O stretching of the protonated propionic side chain in Agp2 (Pfr, Lumi-F). However, a protonated propionic side chain is quite unusual in prototypical phytochromes.^50^ Moreover, such an assignment can be ruled out since none of the IR difference spectra of the Pr → Pfr photoconversion reveals positive or negative signals in this region (SI, Fig. S5). Thus, we tentatively assign these bands to the C=O stretching of ring A, implying that also Pr exists in two sub-states differing with respect to the conformations or hydrogen bonding interactions of C=O groups (Table 1).

### 3.2. The photoreaction of Pfr to Lumi-F in Agp2 and identification of Lumi-F sub-states

Cryogenic IR difference spectroscopy of Agp2 in H_2_O was carried out between 4 and 130 K, whereas in D_2_O no difference signals were obtained at temperatures below 130 K. On the other hand, at temperatures above 130 K in both H_2_O and D_2_O, a secondary photoproduct Pfr′, is formed upon irradiating Lumi-F,^56^ and thus interferes with the generation of a pure Lumi-F/Pfr difference spectrum (SI, Fig. S1 and S4).

**Fig. 4:**
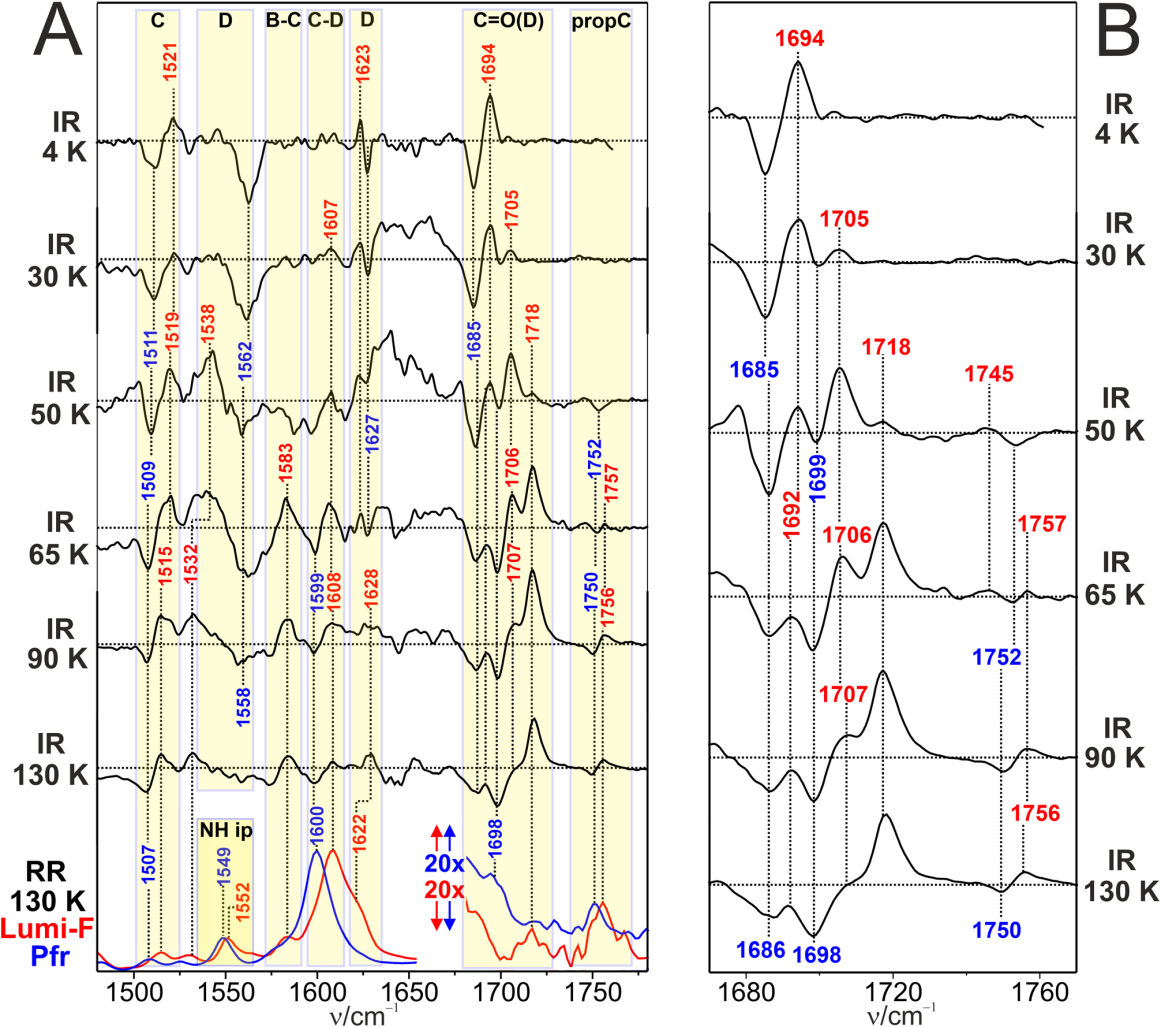
IR difference and RR spectra of Agp2. (A) Cryogenic IR difference spectra in the temperature range from 4 to 130 K (black traces) compared with the RR spectra of Pfr (blue trace) and Lumi-F (red trace) at 130 K. All spectra were measured from Agp2 in H_2_O. Blue and red labels refer to Pfr and Lumi-F, respectively. The yellow-shaded areas indicate the range of the C=O stretching modes (Table 1) and specific BV modes according to previous assignments.^19,45–47^ (B) Close-up view of the C=O stretching region of the IR difference spectra in (A). The horizontal dotted lines in the IR difference spectra mark the intensity difference of zero.

At 4 K, the difference spectrum displays several signal pairs that can readily be attributed to BV, indicating photoconversion at such low temperature (Fig. 4). In the C=O stretching region, we note a signal pair at 1694(+)/1685(−) cm^−1^ originating from the ring D C=O stretching. No signal due to the propC side chain above 1740 cm^−1^ is observed. Upon raising the temperature to 30 K the intensity of the positive 1694 cm^−1^ peak seems to decrease in favor of a band at 1705 cm^−1^, pointing to conformational or environmental change of the C=O(D) group. At 50 K, the band at 1705 cm^−1^ further increases, accompanied by the appearance of a positive signal at 1718 cm^−1^. From 65 to 130 K, the C=O(D) signals of Lumi-F undergo an intensity redistribution such that the 1718 cm^−1^ band finally prevails and the 1692 and 1707 cm^−1^ have nearly disappeared.

The variation of the C=O stretching signals of ring D with the temperature reflects a temperature-dependent distribution of different sub-states. They are denoted as L1, L2, and L3, characterized by the bands at 1694, 1705, and 1718 cm^−1^, respectively. We have chosen this nomenclature in analogy to the work by Yang *et al.*, who identified three Lumi-F sub-states in a temperature-dependent cryo-crystallographic study on the bathy phytochrome PaBphP from *Pseudomonas aeruginosa*,^55^ which is closely related to Agp2.^44,51,52,57^ Hence, we now compare the main findings on Lumi-F sub-state structures in PaBphP^55^ with the spectroscopic results for the corresponding sub-states in Agp2. In sub-state L1 of PaBphP, the structural changes are restricted to ring D and its C=O substituent, which upon rotation around the C–D double bond has lost its hydrogen-bonding partner, a water molecule.^55^ This observation is largely consistent with the IR difference spectra of L1(Agp2) (*e.g.*, 4 K; Fig. 4). The ring D C=O is shifted up from 1685 cm^−1^ (Pfr) to 1694 cm^−1^ in L1 reflecting the loss of coupling of the C=O bond to the delocalized π-electron system BV and a weakening of the hydrogen bonding interactions. Also the other signals of the chromophore are due to modes localized at the isomerization site. The 1623 cm^−1^ band is readily assigned to a C=C stretching mode of ring D and its vinyl substituent.^47^ Furthermore, the negative signal at 1562 cm^−1^ originates from C=C/C–C stretching of ring D of Pfr. Its positive counterpart cannot be identified. It might be obscured by the 1562 cm^−1^ signal. However, in addition, a distinct signal pair at 1521(+)/1511(−) cm^−1^ is observed which falls into the range expected for a ring C stretching mode.^45,46^ This assignment implies that already in L1 structural changes also include ring C, in contrast to the findings by Yang *et al.*^55^ Instead, the crystallographic data indicate the extension of structural changes to ring C only in L2. In fact, in the corresponding IR spectra between 50 and 65 K, dominated by L2, we identify further signals at 1607 and 1583 cm^−1^, which originate from the C–D and B–C stretching mode, respectively.^47^ In L3, the structural changes of the tetrapyrrole skeleton extend to ring A and B.^55^ A specific spectral marker band is the A–B stretching which is expected at *ca.* 1620 cm^−1^ but can hardly be detected due to the overlap with the ring D stretching.

The IR spectra of L2 and L3 both reveal spectral changes that reflect modified hydrogen bonding interactions of the propC and C=O(D) substituents. As suggested by the crystallographic data of Agp2 and PaBphP,^6,55^ in these two sub-states the hydrogen bonding network in the environment of ring C and D occurs, involving Tyr205, Tyr165, His278 (Agp2 numbering), and a conserved water molecule. These are the candidates for interacting with the protonated side chain of propC, weakening the hydrogen bonding interactions from L2 (1745 cm^−1^) to L3 (1757 cm^−1^). Similarly, also the strength of hydrogen bonding interactions of C=O(D) is reduced from L2 (1706 cm^−1^) to L3 (1718 cm^−1^).

On the whole, the low temperature IR spectra are consistent with the structural changes detected by cryo-crystallography.^55^ Deviations refer to the temperature range in which the three sub-states prevail. This may mainly be due to different kinetics of the relaxation processes in the crystalline and in the solid solution state. In line with the crystallographic approaches,^6,55^ there are no protein backbone alterations that would be reflected by signals in the amide I region.

We finally would like to comment on the Pfr signals in the difference spectra. In general, the frequencies of these signals are largely constant in the entire temperature range. Minor deviations by ±2 cm^−1^ can be rationalized in terms of the inherent uncertainty in determining absolute peak positions in difference spectra. Consequently, the shift of the (negative) C=O(D) signal from 1685 cm^−1^ (Pfr-I) to 1698 cm^−1^ (Pfr-II) at 65 K, corresponding to the transition temperature of L2 to L3, suggests that (i) the Pfr sub-states have different photochemical activity, and (ii) with increasing temperature photoconversion occurs predominantly *via* Pfr-II.

### 3.3. The photoreaction of Pr to Lumi-R in Agp1 and identification of Lumi-R sub-states

**Fig. 5:**
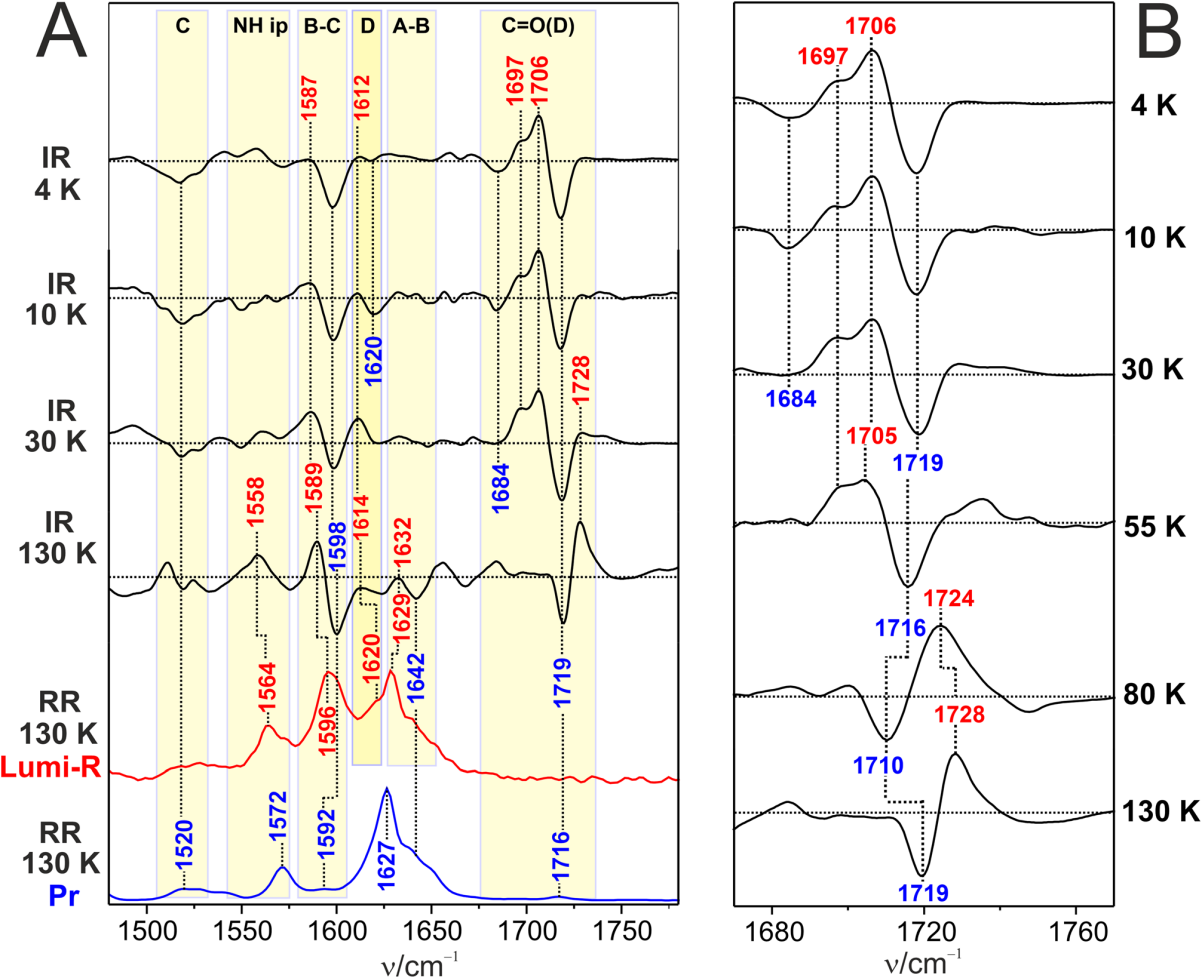
IR difference and RR spectra of Agp1. (A) Cryogenic IR difference spectra in the temperature range from 4 to 130 K (black traces) compared with the RR spectra of Pr (blue trace) and Lumi-R (red trace) at 130 K. All spectra were measured from Agp1 in H_2_O. The blue and red labels refer to Pr and Lumi-R, respectively. The yellow-shaded areas indicate the range of the C=O stretching modes (Table 1) and specific BV modes according to previous assignments.^19,45–47^ (B) Close-up view of the C=O stretching region of IR difference spectra measured at different temperatures from 4 K to 130 K. The horizontal dotted lines in the IR difference spectra mark the intensity difference of zero.

Like the Pfr state of Agp2, the Pr state of Agp1 is photoactive even at 4 K (Fig. 5). At such a low temperature we note pronounced difference signals in the C=O(D) stretching region (right panel) with a strong negative peak at 1719 cm^−1^ and a weaker one at 1684 cm^−1^ that are due to Pr ground state. The positive counterparts involve the main signal at 1706 cm^−1^ with a shoulder at 1697 cm^−1^.

Following the same approach as for Agp2, we first focus on this spectral region to identify different Lumi-R sub-states in Agp1, assuming that each sub-state is characterized by a single specific C=O(D) stretching mode. Accordingly, we assume that two sub-states L1 (1697 cm^−1^) and L2 (1706 cm^−1^) are generated at 4 K and remain at a constant ratio up to 30 K. At this temperature, the third sub-state L3 with a band at 1728 cm^−1^ starts to evolve and becomes the prevailing sub-state at 80 K. The high frequency of this mode suggests a weak electronic coupling to the delocalized π-electron system of BV, which in turn accounts for the lack of any resonance enhancement. Thus, this mode is not detected in the RR spectrum of the Lumi-R L3 sub-state. In the L1 and L2 states, signal pairs in the region of the tetrapyrrole modes are observed at 1612(+)/1620(−) and 1587(+)/1598(−) cm^−1^, which originate from the ring D and B–C stretching modes, respectively. An additional negative signal without a positive counterpart is detected at 1520 cm^−1^, attributed to a ring C stretching mode. Most likely, the Pr and Lumi-R modes strongly overlap such that the latter positive signal is obscured. We therefore conclude that, similar to Agp2, the primary structural changes of the chromophore are localized in the C–D unit but not restricted to ring D.

**Fig. 6:**
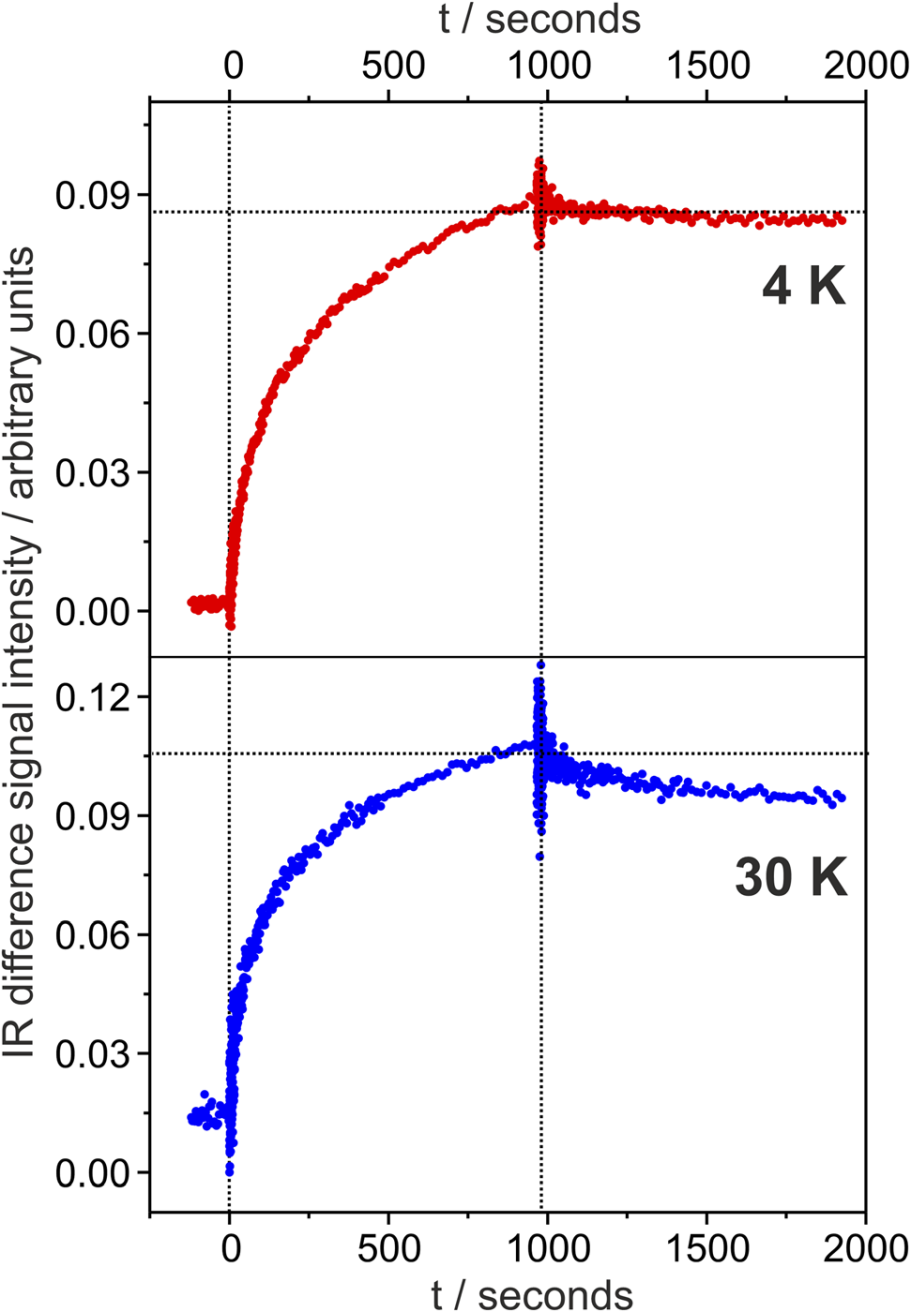
Temporal evolution of the IR difference signal in the C=O stretching region during the irradiation period (0 to *ca.* 1000 s) and the subsequent dark period (*ca.* 1000–2000 s). The data refer to the difference spectra of Pr of Agp1 at 4 K (top) and 30 K (bottom) (see Fig. 5).

In contrast to bathy phytochromes, there are no temperature-scan cryo-crystallographic studies on Agp1 that would allow detecting Lumi-R sub-states. However, we may refer to structural data from X-ray free laser (XFEL) crystallographic investigations on the Pr photoreaction of Agp1-related prototypical bacteriophytochromes in this context. Whereas some of the studies refer to the excited state (GAF-PAS fragment of DrBphP from *Deinococcus radiodurans* and SaBphP2 from *Stigmatella aurantiaca*),^23,25^ the work of Carrillo *et al.* has captured also a snapshot at 5 ns,^22^ which is most likely related to the sub-states L1–L3. This data set demonstrates that the structural changes initiated by ring D rotation are tightly coupled to distortions of ring C and the adjacent methine bridges. These findings are fully in line with the spectral changes in L1 and L2. In addition, at 5 ns also the A–B unit is affected and the pyrrole water is released from the chromophore binding pocket. These two events can readily be correlated with the spectrum of L3 at 130 K, *i.e.* the signal pair of the A–B stretching at 1632(+)/1642(−) cm^−1^ and the positive signal at 1558 cm^−1^ due to the N–H in-plane bending of rings B and C. The XFEL data of DrBphP and SaBphP2 further reveal a reorganization of the hydrogen bonding network involving the propionate side chains of ring B and C and the carbonyl function of ring D as well as the amino acid side chains. With regard to Agp1, these residues are Arg244, Ser262, Ser264, His260, His280, Tyr253, and Asp197, as well as several involved water molecules.^58^ The water-mediated or direct hydrogen bonding interactions of C=O(D) and His280 must be broken with the rotation of ring D (*i.e.*, already in L1 and L2) and replaced by another hydrogen bond donor, as reflected by the relatively low C=O stretching frequency. Further rearrangement of the hydrogen bonded network weakens the hydrogen bonding interactions as shown by the frequency upshift to 1728 cm^−1^. We further tested the thermal stability of the photoproducts L1 and L2 characterized by the signal pair 1706(1697)(+)/1719(−) cm^−1^. The time-dependent changes of this difference signal were monitored at 4 and 30 K, starting with the irradiation period of 15 min and followed by a dark phase of 15 min (Fig. 6). At 4 K, there is only a weak intensity decay to *ca.* 97% during this period of time whereas at 30 K a slightly faster decrease to 90% is observed. Based on these findings, one may estimate the lifetime of L1/L2 at 4 and 30 K to *ca.* 5 and 2.3 hours. Assuming the validity of the Arrhenius equation in this temperature range, this would correspond to an activation energy of *ca.* 25 J mol^−1^ for the transition L1/L2 → L3. Accordingly, we estimate a transition temperature of *ca.* 80 K at which the sub-states L1/L2 and L3 exist in equal portions. This estimate agrees well with the experimental spectra in Fig. 5.

We now ask for the correlation between structural heterogeneity and photochemical activity in Agp1. The negative C=O(D) signals refer to the sub-states of Pr that undergo a photochemical reaction. This is the sub-state Pr–I (1719–1716 cm^−1^) which is active in the entire temperature range. The second sub-state is characterized by a band at 1695 cm^−1^ which was identified in the Pfr/Pr difference spectrum reported previously.^50^ This band is not detectable in the Lumi-R/Pr difference spectra due to the overlap with a positive L1/L2 band. Instead a negative band at 1684 cm^−1^ is identified up to 10 K, which may result from a third sub-state. Alternatively, it corresponds to the 1695 cm^−1^ species and the frequency just shows a steady upshift in the temperature range from 4 to 293 K. In any case, except for the small temperature range up to 10 K, the photochemical reaction appears to originate exclusively from Pr–I.

### 3.4. Comparison with the photoreactions at ambient temperature

Trapping intermediate states by cryogenic spectroscopy relies upon energy barriers that can only be overcome at certain temperatures. In this way, low-temperature IR and RR spectroscopy probe intermediate states that exist in local minima along a reaction pathway. This is a fundamental difference compared to time-resolved (TR) IR spectroscopy at ambient temperature, in which the thermal energy is usually sufficient to overcome barriers of the ground state reactions. Hence, pump–probe spectra at specific delay times do not reflect an intermediate state at an energetic minimum but a snapshot of a distribution of conformations formed during the reaction pathway. These conformations include, but are not restricted to, intermediates of local minima. Thus, vibrational signatures of TR and cryogenic IR spectra may show similarities for specific delay times and trapping temperatures, respectively.

In the electronic excited state of Pfr of Agp2, the relaxation pathway splits into a productive branch leading to Lumi-F, and in an unproductive decay back to the Pfr ground state. This process has been recently studied by TR IR spectroscopy.^35^ At delay times longer than 4 ps, the reaction channel to Lumi-F prevails and two positive signals of the early ground state photoproduct can be identified. These bands at 1735 and 1695 cm^−1^, obtained from Agp2 in D_2_O, are readily assigned to the C=O stretching of propC and ring D, respectively. Unfortunately, attempts to measure IR difference spectra in D_2_O below 90 K failed. Nevertheless, one may safely relate the 1735 cm^−1^ band to the corresponding mode in L2(H_2_O) since the alternative assignment to L3 would imply a H/D downshift by more than 20 cm^−1^, which is highly unlikely (Fig. 4 and Table 1). The C=O stretching mode of ring D at 1695 cm^−1^ in the transient IR spectrum may correspond to L1 (no H/D effect) or L2 (10 cm^−1^ H/D downshift). Regardless of the assignment, at no delay time do the transient IR spectra of the early Lumi-F, reported by Yang *et al.*,^35^ display a vibrational band pattern that fits to one of the cryogenically trapped sub-states. Thus, we conclude that, if the thermal energy is sufficiently high as in the present TR IR spectroscopic experiments, the molecular motions associated with the interaction changes of C=O(D) and propC are largely independent from each other.

## 4. Conclusions

In both Pr (Agp1) and Pfr (Agp2), the primary photoprocesses do not require additional thermal activation since they take place even at 4 K. The primary structural changes are restricted to the isomerization site. Due to the rotation of ring D, the carbonyl substituent changes its environment and thus is a sensitive probe for the conformational relaxation processes that follow photoisomerization. This justifies the use of the C=O(D) stretching as a marker for sub-states that can cryogenically be trapped.

The present study demonstrates that the structural changes of ring C and D are coupled and occur in the sub-states L1 and L2, whereas further structural changes extending to the remainder of the tetrapyrrole take place in L3. On the level of L2 and L3, there are notable structural changes of the amino acids in the chromophore binding pocket which are reflected by the vibrational modes of the ring C and D substituents. However, the IR spectra rule out even small changes of the peptide bonds, as indicated by the lack of any amide I signals in the IR difference spectra at 130 K. The energy barriers between the sub-states are very low, *e.g. ca.* 25 J mol^−1^ for the transition between L1/L2 and L3 during the relaxation of Lumi-R. This is comparable to the thermal energy at very low temperatures but *ca.* 100 times lower at ambient temperature. Therefore, the various sub-states of the Lumi intermediates can only be detected in cryo-IR spectroscopy at sufficiently low temperatures, whereas TR IR spectroscopy monitors the temporal evolution of specific conformational coordinates.

Low energy barriers between sub-states most likely also govern the sub-state equilibria of the parent states. On the basis of the C=O(D) stretching modes, two sub-states were identified for both Pfr and Pr. These sub-states exhibit different photochemical activity. Only at very low temperatures with hindered conformational equilibration (cryo-IR spectroscopy), photoconversion preferentially proceeds *via* a specific sub-state, whereas at ambient temperature (TR IR spectroscopy) ground heterogeneity of the parent states has no impact on the photoprocesses.^20^

## Author contributions

Galaan Merga, Maximilian Große, Patrick Piwowarski: IR spectroscopic measurements; Anastasia Kraskov, Francisco Velazquez Escobar: RR spectroscopic measurements; Norbert Michael, Manal Ebrahim, Luisa Sauthof: protein expression and purification; Patrick Scheerer: funding acquisition, review & editing, supervision, project administration; Franz Bartl, Peter Hildebrandt: conceptualization of idea, funding acquisition, writing, supervision, project administration.

## Conflicts of interest

The authors declare no conflicts of interest.

## Supplementary Material

RA-015-D5RA08731G-s001

## Data Availability

All data that support the findings of this study is included in the manuscript and the accompanying supplementary information (SI). The data is available from the corresponding author. No additional data sets were generated or deposited. SI presenting further RR and IR spectra is accessible *via* DOI: https://doi.org/10.1039/d5ra08731g. Supplementary information is available. See DOI: https://doi.org/10.1039/d5ra08731g.
