# Supplementary material for: Photoisomerization of phytochrome's chromophore: a vibrational spectroscopic view on the primary ground state processes
Source: RSC Adv. 2025 Dec 15;15(58):49889–99. doi: 10.1039/d5ra08731g (PMC12703697; doi:10.1039/d5ra08731g)
Supplement: RA-015-D5RA08731G-s001 [file RA-015-D5RA08731G-s001.pdf]

Supplementary Information (SI) for RSC Advances.
This journal is © The Royal Society of Chemistry 2025

Supporting Information

Photoisomerization of phytochrome´s chromophore: A vibrational spectroscopic view on the primary ground state processes.

Galaan Merga,[1] Maximilian Große,[1] Patrick Piwowarski,[1] Anastasia Kraskov,[2] Francisco Velazquez Escobar,[2] Norbert Michael,[2] Manal Ebrahim,[3] Luisa Sauthof,[3] Patrick Scheerer,[3] Franz Bartl,[1*] Peter Hildebrandt[2*]

[1] Humboldt- Universität zu Berlin, Institut für Biologie, Biophysikalische Chemie, Invalidenstr 42, D-10115 Berlin, Germany

[2] Technische Universität Berlin, Institut für Chemie, Sekr. PC14, Straße des 17. Juni 135, D-10623 Berlin, Germany

[3] Institute of Medical Physics and Biophysics, Group Structural Biology of Cellular Signaling Charité − Universitätsmedizin Berlin, corporate member of Freie Universität Berlin and Humboldt-Universität zu Berlin, Charitéplatz 1, D-10117 Berlin, Germany

[*] Correspondence addresses: Peter.Hildebrandt@TU-Berlin.de; Franz.Bartl@HU-Berlin.de

Resonance Raman spectra of Agp2

RR spectra were measured of Agp2 in the Pfr, Lumi-F, and Pfr´ states as highlighted in the photocycle in Figure S1. In the three states, H/D exchange of the ring D N-H is impaired such that a selected H/D substitution at rings A, B, C vs. ring D is possible.[1,2]

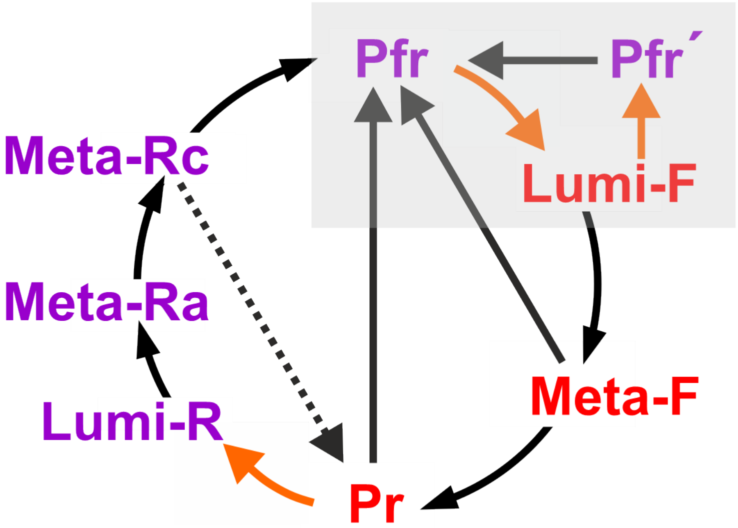

Figure S1. Photocycle of Agp2 and Agp1 with the states in ZZEssa configuration in violet and the states in ZZZssa configuration in red.[3] The orange and black arrows indicate photochemical and black reactions, respectively. The black dotted line represents a reaction that is very likely but not yet confirmed. The gray rectangle highlights those states of Agp2, the RR spectra of which are shown in Figures S2 – S4. The Pfr´ state has been so far only detected for Agp2.

1. RR spectra of Pfr

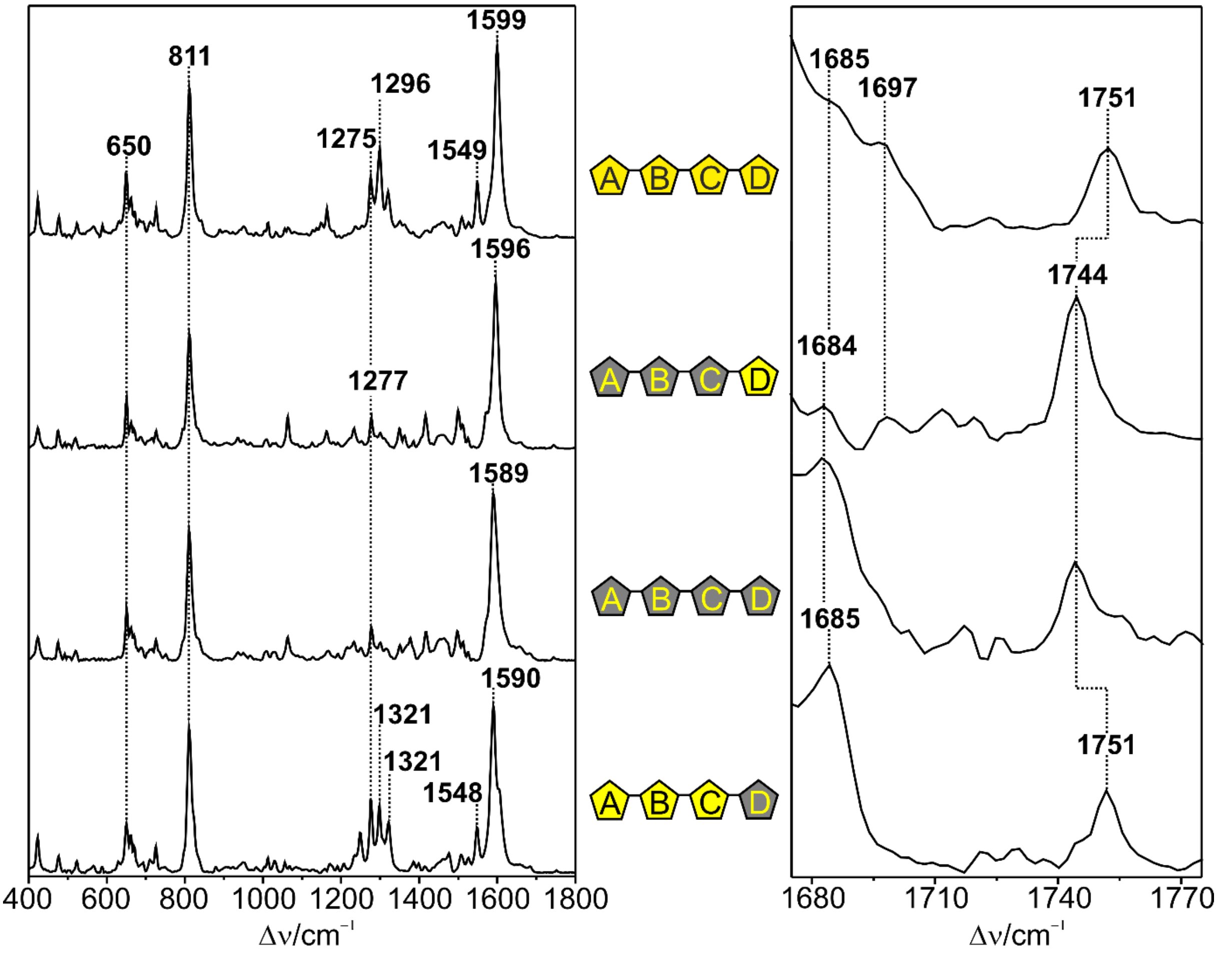

Figure S2. RR of Pfr with different H/D patterns at the pyrrole rings A, B, C vs. D, the ring C protonated propionic side chain. The frequencies in the C=O stretching region (right panel) agree very well with the IR data and thus confirm the assignments given in Table 1 of the manuscript.

2. RR spectra of Lumi-F

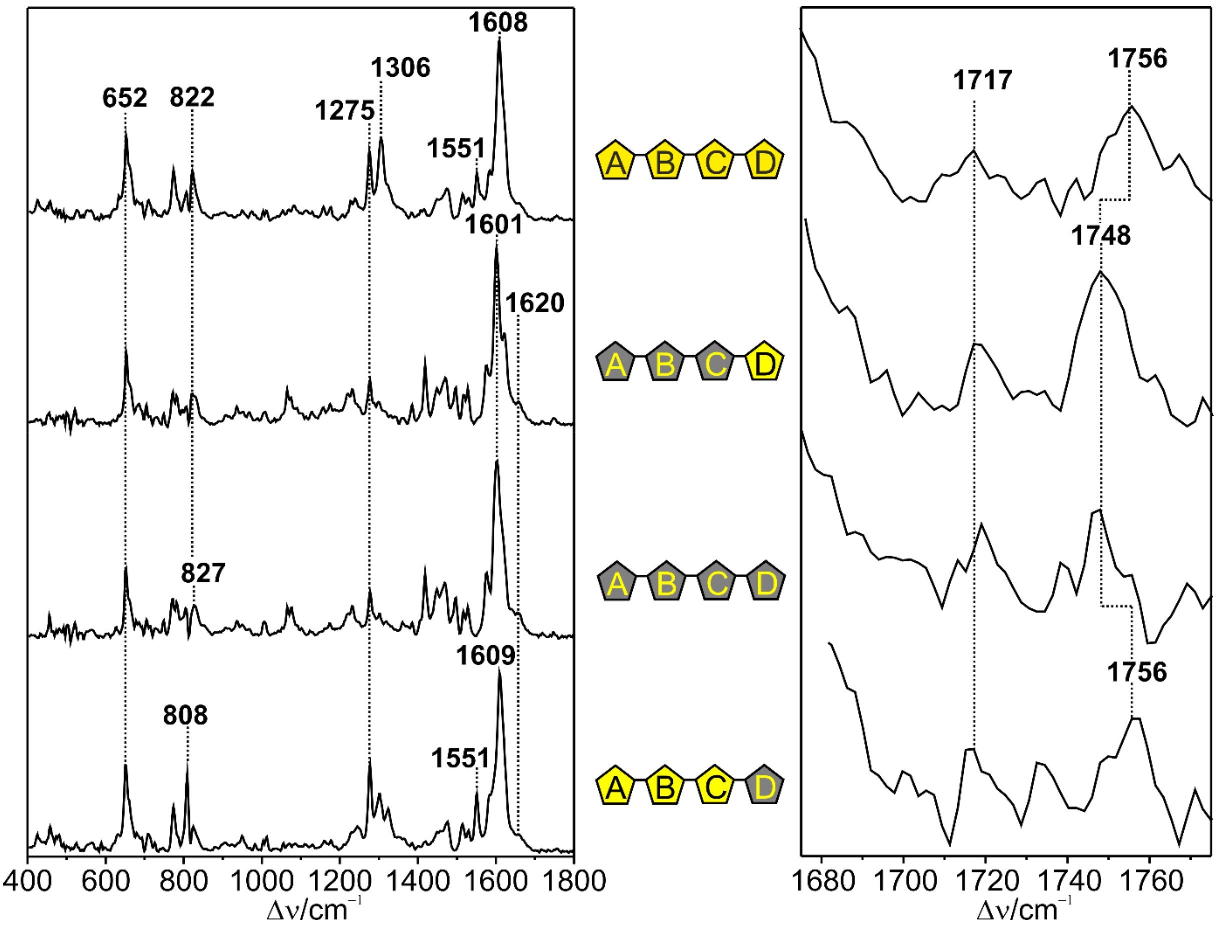

Figure S3. RR of Lumi-F with different H/D patterns at the pyrrole rings A, B, C vs. D, the ring C protonated propionic side chain. The frequencies in the C=O stretching region (right panel) agree very well with the IR data and thus confirm the assignments given in Table 1 of the main paper.

3. RR spectra of Pfr´

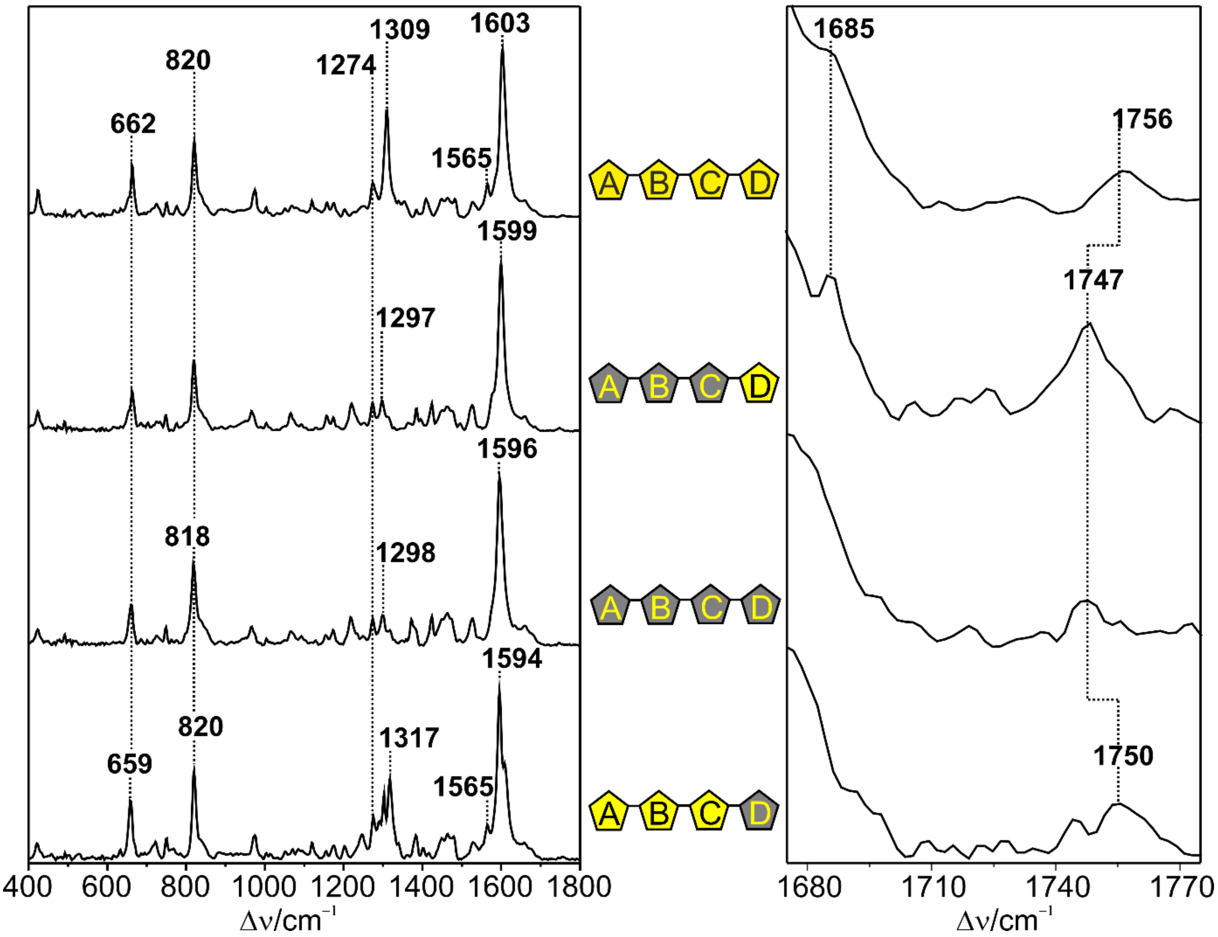

Figure S4. RR of Pfr´ (see Figure S1) with different H/D patterns at the pyrrole rings A, B, C vs. D, the ring C protonated propionic side chain.

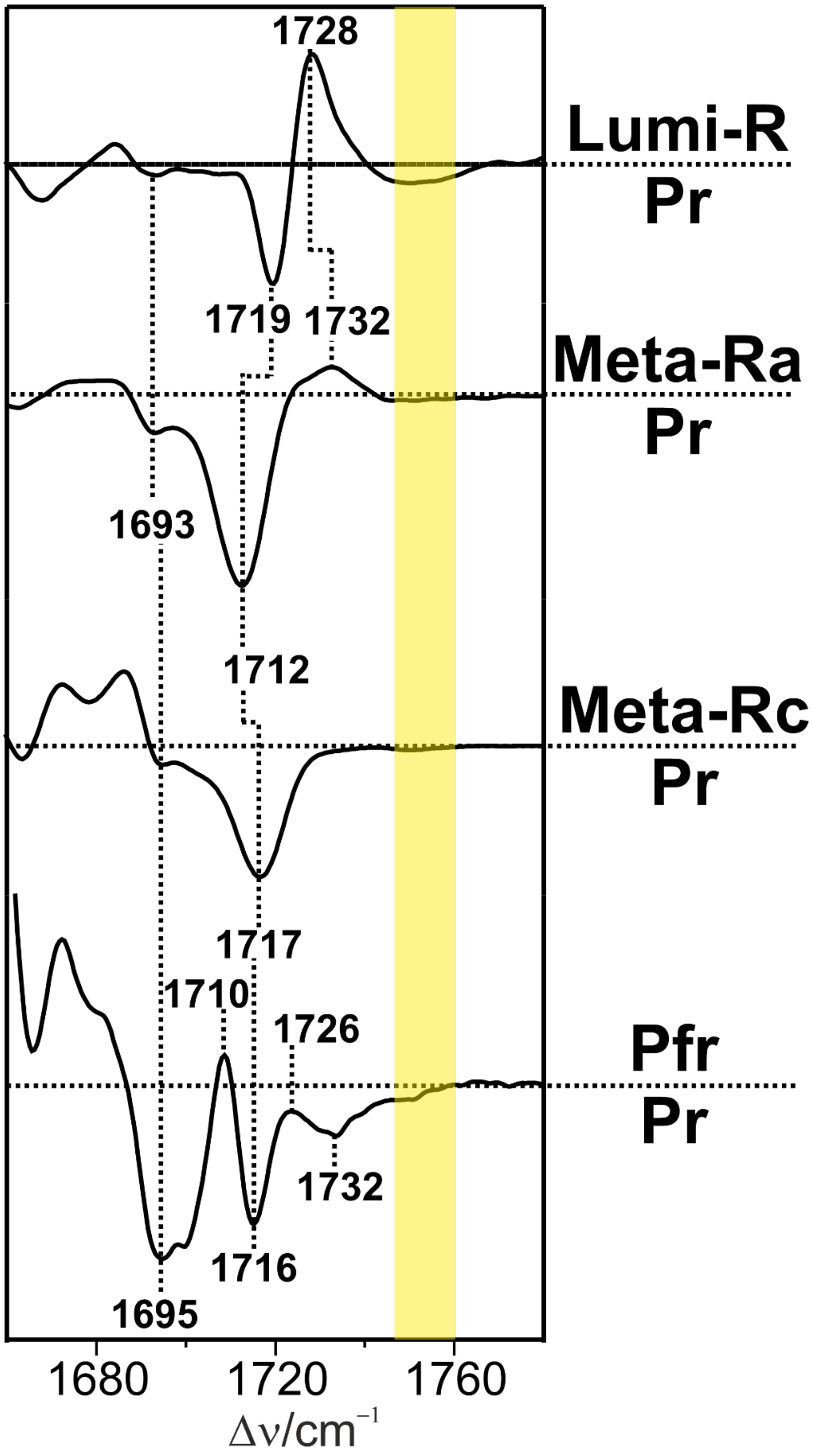

Figure S5. IR difference spectra of the Pr → Pfr reaction pathway of Agp1 in H_2O (see Figure S1) in the C=O stretching region. The yellow rectangle marks the region expected for a protonated propionic side chain, show no indication for a negative or positive signal. The horizontal dotted lines in the IR difference spectra mark the intensity difference of zero.

References

1 F. Velázquez Escobar, D. Buhrke, N. Michael, L. Sauthof, S. Wilkening, N. N. Tavraz, J. Salewski, N. Frankenberg-Dinkel, M. A. Mroginski, P. Scheerer, T. Friedrich, F. Siebert and P. Hildebrandt, *Photochem. Photobiol.*, 2017, **93**, 724–732.

2 F. Velazquez Escobar, P. Piwowarski, J. Salewski, N. Michael, M. Fernandez Lopez, A. Rupp, M. B. Qureshi, P. Scheerer, F. Bartl, N. Frankenberg-Dinkel, F. Siebert, M. A. Mroginski and P. Hildebrandt, *Nat. Chem.*, 2015, **7**, 423–430.

3 P. Hildebrandt, *Biomolecules*, 2023, **13**, 1007.
